# A novel approach for feline sporotrichosis pathogen detection based on loop‐mediated isothermal amplification

**DOI:** 10.1111/vde.13345

**Published:** 2025-04-21

**Authors:** Steffanie Amadei, Júlia Campos, Amanda Bertão‐Santos, Alis Frentzel, Hugo Ávila, Fabiana S. Monti, Marconi R. Farias

**Affiliations:** ^1^ Integrated Program in Neuroscience McGill University Montreal Canada; ^2^ Department of Veterinary Medicine, School of Medicine and Life Sciences Pontifical Catholic University of Paraná Curitiba Brazil; ^3^ Postgraduate Program in Animal Science Pontifical Catholic University of Paraná Curitiba Brazil; ^4^ Department of Bioprocess Engineering and Biotechnology Federal University of Paraná Curitiba Brazil; ^5^ Department of Biotechnology, School of Medicine and Life Sciences Pontifical Catholic University of Paraná Curitiba Brazil

**Keywords:** LAMP loop‐mediated isothermal amplification, mycoses, *Sporothrix*

## Abstract

**Background:**

Sporotrichosis is a chronic, mycotic infection caused by fungi of the genus *Sporothrix*. Zoonotic sporotrichosis occurs mainly through *S. brasiliensis* transmission, resulting from the organism's traumatic introduction via scratches or bites, or contact with exudate from contaminated cats. The loop‐mediated isothermal amplification (LAMP) assay is a viable molecular alternative for detecting *Sporothrix* in veterinary low‐resource settings.

**Hypothesis/Objectives:**

To develop a LAMP method for *Sporothrix* identification using fungal isolates and clinical samples of domestic cats (*Felis catus*).

**Materials and Methods:**

DNA samples were collected from *Sporothrix* isolates and clinical samples of cats positive for sporotrichosis. Six LAMP primers were designed to amplify the 28S ribosomal RNA region of *S. schenckii* and *S. brasiliensis*. Colorimetric assay and agarose gel electrophoresis were used to analyse the LAMP amplification. Isolated samples were sequenced using the Sanger technique, employing the amplification of genetic material by conventional PCR with the external primers of LAMP.

**Results:**

A sensitivity of 96.77% for isolated *Sporothrix* samples was found using the LAMP method, confirmed by Sanger sequencing. The detection limit of LAMP was between 1 and 10 pg of *Sporothrix* DNA according to the sample matrix. LAMP showed a sensitivity of 100% using blood samples, 77.78% using intranasal swabs and 92.31% and 100% using swab and adhesive tape samples of cutaneous lesions, respectively.

**Conclusions and Clinical Relevance:**

These findings support a simple and quick LAMP‐based screening tool for detecting *Sporothrix* in isolated and clinical samples. This accessible test can aid in disease management when standard culture analysis is unavailable or impractical.

## Graphical abstract

**Background –** Sporotrichosis is a chronic, mycotic infection caused by fungi of the genus *Sporothrix*. Zoonotic sporotrichosis occurs mainly through *S. brasiliensis* transmission, resulting from the organism's traumatic introduction via scratches or bites, or contact with exudate from contaminated cats. The loop‐mediated isothermal amplification (LAMP) assay is a viable molecular alternative for detecting *Sporothrix* in veterinary low‐resource settings. **Hypothesis/Objectives –** To develop a LAMP method for *Sporothrix* identification using fungal isolates and clinical samples of domestic cats (*Felis catus*). **Conclusions and Clinical Relevance –** These findings support a simple and quick LAMP‐based screening tool for detecting *Sporothrix* in isolated and clinical samples. This accessible test can aid in disease management when standard culture analysis is unavailable or impractical.

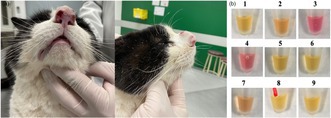

## Zusammenfassung

**Hintergrund:**

Die Sporotrichose ist eine chronische, mykotische Infektion, die durch den Fungus der Gattung *Sporothrix* verursacht wird. Eine zoonotische Sporotrichose tritt vor allem durch eine Übertragung von *S. brasiliensis* auf, resultierend aus einer traumatischen Übertragung des Organismus durch Kratzer oder Bisse, oder dem Kontakt mit Exsudat von kontaminierten Katzen. Die isothermische Nukleinsäure‐Amplifikationstechnik (LAMP) ist eine wertvolle molekulare Alternative zur Bestimmung von *Sporothrix* in einem Veterinärsetting mit wenig Ressourcen.

**Hypothese/Ziele:**

Die Entwicklung einer LAMP‐Methode zur *Sporothrix* Identifizierung mittels Fungus Isolaten und klinischen Proben von Hauskatzen (*Felis catus*).

**Materialien und Methoden:**

Es wurden DNA‐Proben von *Sporothrix* Isolaten und von klinischen Proben von Sporotrichose‐positiven Katzen gesammelt. Sechs LAMP‐Primer wurden designed, um die 28S ribosomale RNA‐Region von *S. schenkii* und *S. brasiliensis* zu amplifizieren. Ein kolorimetrischer Assay und eine Agarose‐Gelelektrophorese wurden eingesetzt, um die LAMP‐Amplifizierung zu analysieren. Die isolierten Proben wurden mittels Sanger Technik sequenziert, wobei die Amplifizierung von genetischem Material mittels konventionellem PCR mit den externen Primern des LAMP durchgeführt wurde.

**Ergebnisse:**

Für die isolierten *Sporothrix* Proben wurde mittels LAMP‐Methode eine Sensibilität von 96,77% gefunden, was durch Sanger Sequenzierung bestätigt wurde. In Bezug auf die Probenmatrix lag das Nachweislimit durch LAMP zwischen 1 und 10 pg von *Sporothrix* DNA. Die LAMP zeigte bei Verwendung der Blutproben eine Sensibilität von 100%, bei Verwendung der intranasalen Tupfer 77,78% und 92,3% bzw 100% bei den Tupfern bzw Klebestreifenproben der kutanen Läsionen.

**Schlussfolgerungen und klinische Bedeutung:**

Diese Ergebnisse unterstützen eine einfache und schnelle LAMP‐basierte Screening Methode, um *Sporothrix* in isolierten und klinischen Proben nachzuweisen. Dieser zugängliche Test kann beim Management der Krankheit behilflich sein, wenn eine Standardkulturanalyse nicht zur Verfügung steht oder unpraktisch ist.

## 摘要

**背景:**

孢子丝菌病是由孢子丝菌属真菌引起的一种慢性真菌感染。人畜共患孢子丝菌病主要通过巴西孢子丝菌传播，该病原体通过抓伤、咬伤或接触受污染猫的渗出液而传入。环介导等温扩增 (LAMP) 检测是一种可行的分子检测方法，可用于在兽医资源匮乏的环境中检测孢子丝菌。

**假设/目标:**

开发一种LAMP方法鉴定孢子丝菌(Sporothrix)，用于识别真菌分离株和家猫(Felis catus)的临床样本，。

**材料与方法:**

从孢子丝菌分离株以及诊断为孢子丝菌病阳性猫的临床样本中提取DNA。设计了六种LAMP引物，用于扩增申克氏孢子丝菌(S. schenckii)和巴西孢子丝菌(S. brasiliensis)的 28S 核糖体 RNA 区。采用比色法和琼脂糖凝胶电泳法分析LAMP扩增结果。分离株通过桑格技术进行测序，该技术利用LAMP的外部引物，通过常规PCR扩增基因物质。

**结果:**

LAMP方法对分离孢子丝菌样本的灵敏度为96.77%，并经桑格测序证实。根据样本基质的不同，LAMP的检测限为1至10 pg孢子丝菌DNA。LAMP方法对血液样本的灵敏度为100%，对鼻腔拭子样本的灵敏度为77.78%，对皮肤病变拭子和胶带样本的灵敏度分别为92.31%和100%。

**结论及临床意义:**

这些研究结果支持一种基于LAMP的简单快速筛查工具，用于检测分离株和临床样本中的孢子丝菌。当无法进行或无法进行标准培养分析时，这种便捷的检测方法有助于疾病管理。

## Résumé

**Contexte:**

La sporotrichose est une infection mycosique chronique causée par des champignons du genre *Sporothrix*. La sporotrichose zoonotique se produit principalement par la transmission de *S. brasiliensis*, résultant de l'introduction traumatique de l'organisme par des griffures ou des morsures, ou par le contact avec l'exsudat de chats contaminés. Le test d'amplification isotherme en boucle (LAMP) est une alternative moléculaire viable pour la détection de *Sporothrix* dans les environnements vétérinaires à faibles ressources.

**Hypothèse/objectifs:**

Développer une méthode LAMP pour l'identification de *Sporothrix* à partir d'isolats de champignons et d'échantillons cliniques de chats domestiques (*Felis catus*).

**Matériels et méthodes:**

Des échantillons d'ADN ont été prélevés sur des isolats de *Sporothrix* et des échantillons cliniques de chats positifs pour la sporotrichose. Six amorces LAMP ont été conçues pour amplifier la région de l'ARN ribosomique 28S de *S. schenckii* et *S. brasiliensis*. Un test colorimétrique et une électrophorèse sur gel d'agarose ont été utilisés pour analyser l'amplification LAMP. Les échantillons isolés ont été séquencés à l'aide de la technique Sanger, en utilisant l'amplification du matériel génétique par PCR conventionnelle avec les amorces externes de LAMP.

**Résultats:**

La méthode LAMP a permis d'obtenir une sensibilité de 96,77 % pour les échantillons isolés de *Sporothrix*, confirmée par le séquençage Sanger. La limite de détection de la méthode LAMP se situait entre 1 et 10 pg d'ADN de *Sporothrix* en fonction de la matrice de l'échantillon. La méthode LAMP a montré une sensibilité de 100 % pour les échantillons de sang, de 77,78 % pour les écouvillons intranasaux et de 92,31 % et 100 % pour les échantillons de lésions cutanées prélevés sur des écouvillons et des rubans adhésifs, respectivement.

**Conclusions et pertinence clinique:**

Ces résultats confirment l'existence d'un outil de dépistage simple et rapide basé sur la méthode LAMP pour détecter *Sporothrix* dans des échantillons isolés et cliniques. Ce test accessible peut contribuer à la prise en charge de la maladie lorsque l'analyse de la culture standard n'est pas disponible ou n'est pas pratique.

## 要約

**背景:**

スポロトリコーシスはSporothrix属の真菌によって引き起こされる慢性の真菌感染症である。人獣共通感染症であるスポロトリコーシスは、主に*S. brasiliensis*の感染によって発症し、ひっかき傷や咬傷、あるいは汚染された猫の滲出液との接触によって、この菌が外傷的に持ち込まれる。LAMP法(loop‐mediated isothermal amplification:ループ媒介等温増幅法)は、獣医療におけるリソースが限られた環境においてSporothrixを検出するための、実行可能な分子学的代替法である。

**仮説/目的:**

本研究の目的は、真菌分離株と家猫(Felis catus)の臨床サンプルを用いて、Sporothrix同定のためのLAMP法を開発することであった。

**材料と方法:**

Sporothrix分離株およびSporothrix症陽性猫の臨床検体からDNAサンプルを採取した。*S. schenckii*および*S. brasiliensis*の28SリボソームRNA領域を増幅する6種類のLAMPプライマーを設計した。LAMP増幅解析には比色測定およびアガロースゲル電気泳動を用いた。分離したサンプルは、LAMPの外部プライマーを用いた通常のPCRによる遺伝物質の増幅を採用し、サンガー法を用いて塩基配列を決定した。

**結果:**

LAMP法を用いた分離Sporothrixサンプルの感度は96.77%であった。LAMP法の検出限界はサンプルマトリックスにより1~10 pgであった。LAMP法の感度は、血液サンプルで100%、鼻腔内スワブで77.78%、皮膚病変のスワブと粘着テープサンプルでそれぞれ92.31%と100%であった。

**結論と臨床的意義:**

これらの知見は、分離サンプルおよび臨床サンプル中のSporothrixを検出するための、簡便かつ迅速なLAMPベースのスクリーニングツールを支持するものであった。この簡便な検査は、標準的な培養検査が利用できない場合や実施できない場合に、疾患の管理に役立つ可能性がある。

## Resumo

**Contexto:**

A esporotricose é uma infecção micótica crônica causada por fungos do gênero *Sporothrix*. A esporotricose zoonótica ocorre principalmente pela transmissão de *S. brasiliensis*, resultante da inoculação traumática do organismo em soluções de continuidade como arranhões ou mordeduras, ou contato com exsudato de gatos contaminados. O ensaio de amplificação isotérmica mediada por alça (LAMP) é uma alternativa molecular viável para a detecção de *Sporothrix* em estabelecimentos veterinários com poucos recursos.

**Hipótese/Objetivos:**

Desenvolver um método LAMP para identificação de *Sporothrix* utilizando isolados de fungos e amostras clínicas de gatos domésticos (*Felis catus*).

**Materiais e Métodos:**

Amostras de DNA foram coletadas de isolados de *Sporothrix* e amostras clínicas de gatos positivos para esporotricose. Seis primers LAMP foram desenvolvidos para amplificar a região 28S do RNA ribossomal de *S. schenckii* e *S. brasiliensis.* Ensaio colorimétrico e eletroforese em gel de agarose foram utilizados para analisar a amplificação de LAMP. Amostras isoladas foram sequenciadas pela técnica de Sanger, empregando a amplificação do material genético por PCR convencional com os primers externos de LAMP.

**Resultados:**

Observou‐se uma sensibilidade de 96,77% para amostras isoladas de *Sporothrix* utilizando o método LAMP, confirmada pelo sequenciamento de Sanger. O limite de detecção de LAMP situou‐se entre 1 e 10 pg de DNA de *Sporothrix*, de acordo com a matriz da amostra. O LAMP apresentou sensibilidade de 100% em amostras de sangue, 77,78% em swabs intranasais e 92,31% e 100% em amostras de swabs e fitas adesivas de lesões cutâneas, respectivamente.

**Conclusões e Relevância Clínica:**

Estes achados corroboram com a utilização de uma ferramenta de triagem simples e rápida baseada em LAMP para a detecção de *Sporothrix* em amostras isoladas e clínicas. Este teste acessível pode auxiliar no manejo da doença quando a análise de cultura padrão não estiver disponível ou for impraticável.

## RESUMEN

**Introducción:**

La esporotricosis es una infección micótica crónica causada por hongos del género *Sporothrix*. La esporotricosis zoonótica se produce principalmente por transmisión de *S*. *brasiliensis*, como resultado de la introducción traumática del organismo a través de arañazos o mordeduras, o del contacto con exudado de gatos contaminados. El ensayo de amplificación isotérmica mediada por lazos (LAMP) es una alternativa molecular viable para la detección de *Sporothrix* en entornos veterinarios de bajos recursos.

**Hipótesis/Objetivos:**

Desarrollar un método LAMP para la identificación de *Sporothrix* utilizando aislados de hongos y muestras clínicas de gatos domésticos (*Felis catus*).

**Materiales y métodos:**

Se obtuvieron muestras de DNA de aislados de *Sporothrix* y muestras clínicas de gatos positivos para esporotricosis. Se diseñaron seis cebadores LAMP para amplificar la región 28S del RNA ribosómico de *S*. *schenckii* y *S*. *brasiliensis*. Se utilizó un ensayo colorimétrico y una electroforesis en gel de agarosa para analizar la amplificación de LAMP. Las muestras aisladas se secuenciaron mediante la técnica de Sanger, empleando la amplificación del material genético mediante PCR convencional con los cebadores externos de LAMP.

**Resultados:**

Se encontró una sensibilidad del 96,77 % para muestras aisladas de *Sporothrix* mediante el método LAMP, confirmada mediante secuenciación de Sanger. El límite de detección de LAMP se situó entre 1 y 10 pg de DNA de *Sporothrix* según la matriz de la muestra. LAMP mostró una sensibilidad del 100 % con muestras de sangre, del 77,78 % con hisopos intranasales y del 92,31 % y del 100 % con muestras de hisopos y cinta adhesiva de lesiones cutáneas, respectivamente.

**Conclusiones y relevancia clínica:**

Estos hallazgos apoyan una herramienta de cribado sencilla y rápida basada en LAMP para la detección de *Sporothrix* en muestras aisladas y clínicas. Esta prueba accesible puede facilitar el manejo de la enfermedad cuando el análisis de cultivos estándar no está disponible o resulta poco práctico.

## INTRODUCTION

Sporotrichosis is a chronic, granulomatous and invasive mycotic infection caused by fungi from the *Sporothrix schenckii* complex. The disease has a wide geographical distribution, occurring mainly in regions with a tropical and subtropical climate, and affects humans, as well as domestic and wild mammals.^1^ *Sporothrix schenckii* (sensu lato) is found in organic and decomposing matter, with human infections historically linked to trauma involving contaminated materials.^2^ By contrast, epidemic outbreaks of urban sporotrichosis in humans have been increasingly linked to feline infections,^3^ which are associated with *Sporothrix brasiliensis* infection. Zoonotic transmission can occur through bites or scratches, or direct contact with lesions on infected cats,^4^, ^5^ which often are unneutered males with free access to the outdoors.^6^, ^7^ The primary forms of feline sporotrichosis can manifest as cutaneous and extracutaneous symptoms. Cutaneous cases may involve single or multiple nodules or isolated or multifocal ulcerative lesions on the skin, while extracutaneous cases may mainly involve respiratory symptoms and swollen lymph nodes. As feline sporotrichosis presents a broad spectrum of clinical signs, the diagnosis relies on laboratory tests.^8^

The standard diagnosis method is fungal culture and morpho‐physiological identification.^9^ Culture is considered the gold standard for sporotrichosis diagnosis, yet is a time‐consuming technique that can generate false‐negative results owing to its susceptibility to contamination and thus delay disease investigation.^10^ Cytopathological investigation can serve as a preliminary diagnostic tool for sporotrichosis. Although cytological assays are rapid, they have lower sensitivity and depend heavily on the parasitic load and observer's expertise. This dependence increases the risk of errors, such as misreading or misidentifying the samples.^11^ Recent progress in the genotyping and identification of emerging species of *Sporothrix* has influenced the study of molecular diagnosis for the disease. As a highly sensitive and specific technique, nearly all studies available for molecular diagnosis of sporotrichosis over the past decades have involved PCR‐based identification.^12^, ^13^, ^14^, ^15^, ^16^ However, molecular techniques are costly and often require adequate laboratory facilities,^17^ making widespread adoption difficult in low‐resource settings.

A potential solution for making molecular testing more accessible is using isothermal amplification assays, simplifying the required instrumentation and reducing assay time.^18^ One such method is loop‐mediated isothermal amplification (LAMP), which offers a significant advantage over traditional PCR. LAMP does not require template denaturation to separate DNA strands because it uses strand‐displacing polymerases. This property eliminates the need for a thermocycler,^19^ making LAMP more affordable and speeding up the amplification process, as it can be performed at a constant temperature. These properties make LAMP an accessible and cost‐effective tool for detecting infectious diseases, such as sporotrichosis, in settings with limited resources. LAMP has been used to detect other feline diseases,^20^, ^21^, ^22^ which demonstrates its reliability and applicability in veterinary diagnostics. Therefore, this study aimed to develop a LAMP assay to assist lesional *Sporothrix* detection in veterinary clinical settings as an alternative rapid and low‐cost molecular screening tool.

## MATERIALS AND METHODS

### Ethics

The Institutional Ethics in Research Committee approved patients' fluid samples collected from 2022 to 2023 under protocol numbers 02237 and 02292. All of the samples were obtained with the cat owner's consent.

### Sample collection

For the specimen study, 31 exudate samples were collected by swabbing lesions with a Stuart medium swab from cats, which confirmed positive for sporotrichosis. The diagnosis was based on combining clinical manifestations and detecting *Sporothrix* by fungal culture performed at the Small Animal Teaching Hospital‐PUCPR. The samples were cultured axenically on Sabouraud's dextrose agar with chloramphenicol (0.05 g/L) at 25 ± 1°C.^9^ After 7 days of growth, *Sporothrix* morphology was characterised by widefield microscopy, staining with lactophenol blue solution.^23^ A single colony was isolated through the quadrant streak method and proceeded for DNA extraction.

Following the specimen study, 46 clinical samples were collected from cats with sporothricosis with respiratory and cutaneous signs and lesion sites. This collection comprised 15 samples of peripheral blood, 13 samples of exudate from cutaneous lesions collected using a Rayon swab, 9 samples of exudate collected from both intranasal nostrils with a nasopharyngeal Rayon swab and 9 samples of exudate from cutaneous lesions collected using the tape‐stripping technique. Additionally, two domestic cats that were negative for sporotrichosis donated peripheral blood to be used in this study.

### DNA extraction

Microbiological isolates were extracted after 7 days of growth using the FastDNA Spin Kit (MP Biomedicals) following the manufacturer's recommendations; this was based on ceramic spheres for cell lysis and purification by a silica‐based method. The fluid's patient samples were extracted using the PureLink Genomic DNA Mini Kit (Invitrogen/Thermo Fisher Scientific) following the manufacturer's procedure. In this, 200 μL of blood was collected, EDTA anticoagulated and stored at −20°C until sample processing. Meanwhile, swabs and adhesive tape samples were stored in a sterile microcentrifuge tube containing 500 μL CLS‐Y fungal analysis solution (MP Biomedicals) and extracted within 48 h of collection. The DNA quantification was determined with a NanoDrop (Thermo Fisher Scientific) spectrophotometer. The extracted DNA quality was determined by the ratio of absorbance at wavelengths of 230, 260 and 280 nm, such as the ratio of absorbance at 260 and 280 nm (the A260/280 ratio) and the A260/230 ratio, according to the manufacturer's instructions. Extracted DNA was stored at −20°C.

### Primer design

Conserved regions in the 28S large subunit ribosomal gene from South America's prevalent species of feline sporotrichosis were analysed for primer design. First, ribosomal sequences were obtained from GenBank (NCBI)^24^ for *S. brasiliensis* (RefSeq assembly: GCF_000820605.1) and *S. schenckii* (RefSeq assembly: GCF_000961545.1). The sequences were aligned using the clustalW Multiple Sequence Alignment tool to identify conserved regions.^25^ These conserved regions were further refined with the gblocks program,^26^ which removes poorly aligned positions and gap regions to improve alignment quality. The refined conserved regions identified with gblocks were used to generate consensus sequences, representing the most conserved portions of the target regions. These consensus sequences provided the foundation for designing LAMP primers.

The primers were designed using primerexplorer v5 software (https://primerexplorer.jp/e/intro/index.html), which evaluates key metrics such as GC content, primer length, melting temperature and the avoidance of secondary structures such as hairpins or dimers. Primers with optimal metrics were ranked, and the top candidates were selected for experimental validation in the laboratory to ensure their specificity and efficiency for detecting *Sporothrix* spp. A set of six specific LAMP primers was then designed: forward inner primer (FIP: 5′‐TCGCCCCAATGCTTGACTCC‐3′), backward inner primer (BIP: 5′‐TGTATGGGTGCCAAACCCGCAC‐3′), forward outer primer (SpR_F3: 5′‐TCCAAGCGGTAAGGCTCTT‐3′), backward outer primer (SpR_B3: 5′‐GCTTCGGCAAGGATGCTG‐3′), forward loop primer (Loop‐F: 5′‐TCCGTGTTTCAAGACGGGT‐3′) and backward loop primer (Loop‐B: 5′‐TGAAAGTAAACGCAGGTGAGAG‐3′).

### 28S target LAMP selectivity in silico

The selectivity of LAMP outer primers (SpR_F3/SpR_B3) targeting the 28S region was tested in silico using the primer‐Blast and nucleotide Blast (blastn) programs.^27^, ^28^ For the primer‐blast analysis, primer specificity was evaluated against nontarget sequences from members of the Felidae family (taxid: 9681) and feline‐associated pathogens. These included *Toxoplasma gondii* (taxid: 5811), *Microsporum canis* (taxid: 63405), *Leishmania* spp. (taxid: 5658), *Blastomyces dermatitidis* (taxid: 5039), *Cryptococcus* spp. (taxid: 5206) and *Histoplasma* spp. (taxid: 5036). Stringency parameters were configured to detect nontarget sequences with fewer than five nucleotide mismatches, including at least two total mismatches and at least two mismatches within the last five nucleotides at the 3′ end. To further validate primer specificity for species not listed by primer‐Blast, the primer was analysed using Blastn. Primer sequences were queried against genomic databases from *Mycobacterium leprae* (GenBank: NZ_CP029543.1), *Mycobacterium tuberculosis* (GenBank: AL123456.3) and *Staphylococcus aureus* (GenBank: AP017922.1). The Blastn algorithm was optimised to identify highly similar sequences.

### LAMP protocol

The LAMP technique^19^ was performed using the WarmStart Colorimetric LAMP 2X Master Mix (New England BioLabs Japan Inc.) following the manufacturer's recommendations, which included 12.5 μL of the master mix (2×), 2.5 μL of the primer mix (10×), 1 μL of target DNA (1 ng/μL of isolated DNA and at variable concentrations for patient sample) and 9 μL of ultrapure water, for a final volume of 25 μL per reaction. The primer mix (10×) included different concentrations of the primer's pairs (FIP/BIP 16 μm, SpR_F3/SpR_B3 2 μm, LOOP‐F/LOOP‐B 4 μm). The amplification conditions were set at 65°C for 30 min, followed by cooling to 16°C in the ProFlex thermocycler (Applied Biosystems). The results were analysed using both the colorimetric indicator and the ladder‐like band pattern produced by LAMP amplification. Visual interpretation of LAMP results was based on the colour change observed after amplification using the colorimetric indicator. A pink colour indicated a negative result for amplification, while a yellow gradient was considered a positive result. The LAMP product was subjected to electrophoresis on a 2% agarose gel, stained with ethidium bromide to confirm the amplicon specificity. All reactions were performed with three technical replicates.

### Sequencing of the outer LAMP primer product

For sequencing, a PCR reaction^29^ was performed using the outer LAMP primers (SpR_F3/SpR_B3). For that, the samples were amplified using 10 μL of Gotaq (2×) PCR master mix reagent (Promega), 2 μL of primer mix (10×), 2 μL of target DNA (1 ng/μL per isolated samples and at variable concentrations for patient sample) and 6 μL of ultrapure water, for a final volume of 20 μL per reaction. The primer mix (10×) consisted of 5 μm of each F3 and B3 primer. PCR amplification cycling conditions were performed in the ProFlex (Applied Biosystems) thermocycler, with an initial denaturation step of 95°C for 10 min, followed by a PCR cycling at 95°C for 15 s and 60°C for 60 s for 50 cycles. The product amplification was analysed using gel electrophoresis, consisting of 1.5% agarose gel in 1× TBE buffer running at 90 V for 60 min. The samples were stained with ethidium bromide, and the single band DNA fragment, resulting in a ~197 bp amplified product, was purified using a QIAquick Gel Extraction Kit (QIAGEN) following the manufacturer's protocol. Eluted material was subjected to sequencing analysis using the Sanger dideoxy sequencing method. The Sanger sequences were analysed using the PHPH electropherogram quality analysis tool^30^, ^31^ based on the CAP3 sequence assembly program.^32^ The sequences were then deposited in GenBank (NCBI).^24^

Sequence similarity was investigated using Blastn ^27^ against *Sporothrix* species. The sequences were compared to genomic databases from *S. brasiliensis* (GenBank: GCF_000820605.1), *S. schenckii* (GenBank: GCF_000961545.1), *Sporothrix globosa* (GenBank: GCA_001630435.1), *Sporothrix luriei* (GenBank: GCA_021398005.1), *Sporothrix pallida* (GenBank: GCA_021396235.1) and *Sporothrix mexicana* (GenBank: GCA_021396375.1). The Blast algorithm was optimised to identify highly similar sequences.

### 28S target LAMP selectivity in vitro

The selectivity of the LAMP primers was further tested by performing a LAMP assay using DNA from *Leishmania infantum, M. leprae, F. catus, Puma concolor, Leopardus wiedii, Leopardus guttulus* and *Leopardus pardalis*. The LAMP assay was conducted as described previously.

### LAMP limit of detection

The lower detection limit (LOD) of the 28S gene sequence detectable by LAMP was evaluated in ultrapure water and spiked samples. In ultrapure water, the LOD was determined using 10‐fold serial dilutions of isolated *Sporothrix* DNA, starting at 10 and ending at 10^−5^ ng. Spiked samples were evaluated using 10‐fold serial dilutions of isolated *Sporothrix* DNA, starting at 1 ng and ending at 10^−4^ ng, in blood‐spiked matrices from sporotrichosis‐negative cats. All reactions were performed with two technical replicates.

### Conventional PCR using feline gene 28S ribosomal DNA

A conventional PCR reaction was performed targeting the feline 28S ribosomal DNA (rDNA) as described by Santos et al.^33^ The PCR was performed using the GoTaq PCR Core System (Promega), including 200 nm of primers forward (Fw: 5′‐AGCAGGAGGTGTTGGAAGAG‐3′) and reverse (Rv: 5′‐AGGGAGAGCCTAAATCAAAGG‐3′) and 3 μL of DNA in a total volume of 25 μL. The positive control samples were blood samples of a cat negative for sporotrichosis, and a negative control with water was included in each run. PCR amplification cycling conditions were performed in the ProFlex (Applied Biosystems) thermocycler, with an initial denaturation step of 95°C for 15 min, followed by a PCR cycling at 95°C for 10 s and 60°C for 30 s for 45 cycles. The product amplification was analysed using gel electrophoresis.

## RESULTS

**FIGURE 1:**
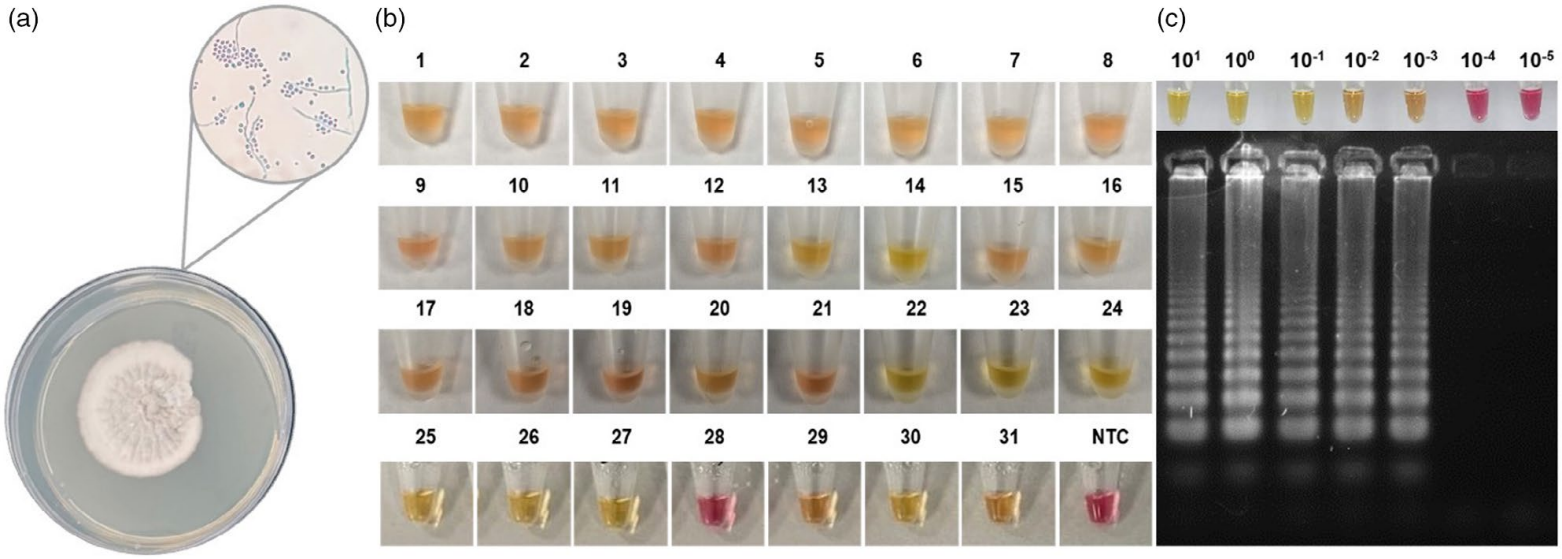
*Sporothrix* identification and loop‐mediated isothermal amplification (LAMP) assay characterisation. (a) *Sporothrix* identification via fungal culture and morphological analysis, represented by a microscopy image of the characteristic flower‐like conidia arrangement. (b) Colorimetric LAMP assay results for *Sporothrix* isolates obtained from cats diagnosed with sporotrichosis. (c) Limit of detection (LOD) for the colorimetric LAMP assay using *Sporothrix* DNA templates (ranging from 10 to 10^−5^ ng) and gel electrophoresis analysis of the assay products. Colour interpretation: pink represents negative reactions, while yellow or orange indicates positive LAMP amplification. NTC, nontarget control.

The LAMP primers (Table 1) did not present significant homology outside the genus of *Sporothrix*, making reliable and specific primers. Thirty‐one isolated samples of *Sporothrix* were analysed using the amplification LAMP method to test the performance of the designed oligonucleotides (Figure 1a). The designed primers amplified 30 of 31 samples (Figure 1b), showing a sensitivity of 96.77%. Then, the isolated samples were subjected to sequencing to confirm that the primers amplified the 28S region. As LAMP products present a cauliflower‐like DNA, owing to the way lamp primers anneal in the target sequence,^19^ it was decided to sequence the PCR product using the SpR_F3/SpR_B3 LAMP primers. Of the 30 isolated amplified samples, 24 presented sufficient DNA and purity for Sanger sequencing deposited at the GenBank database under the accession numbers PP626114 to PP626137. All sample sequences presented 100% identity to the reference genomes of *S. brasiliensis*, *S. globosa, S. globosa*, *S. luriei, S. pallida* and *S. Mexicana* with maximum alignment scores. The selectivity of the 28S LAMP primers was tested with nontarget samples, and no false‐positive amplification was observed. Various feline *Sporothrix* hosts also were tested, and false‐positive results were not observed again. The LAMP assay's LOD for isolates was determined to be 1 pg of *Sporothrix* DNA in ultrapure water (Figure 1c).

**TABLE 1:** Loop‐mediated isothermal amplification (LAMP) primers designed to detect the 28S region of *Sporothrix brasiliensis* and *S. schenckii*.

Primer	Sequence 5′–3′
SpR_F3	TCCAAGCGGTAAGGCTCTT
SpR_B3	GCTTCGGCAAGGATGCTG
FIP	TCGCCCCAATGCTTGACTCCGCGTAATGGTCACCAGCG
BIP	TGTATGGGTGCCAAACCCGCACATCAGGATCGGTCGATGA
Loop‐F	TCCGTGTTTCAAGACGGGT
Loop‐B	TGAAAGTAAACGCAGGTGAGAG

**FIGURE 2:**
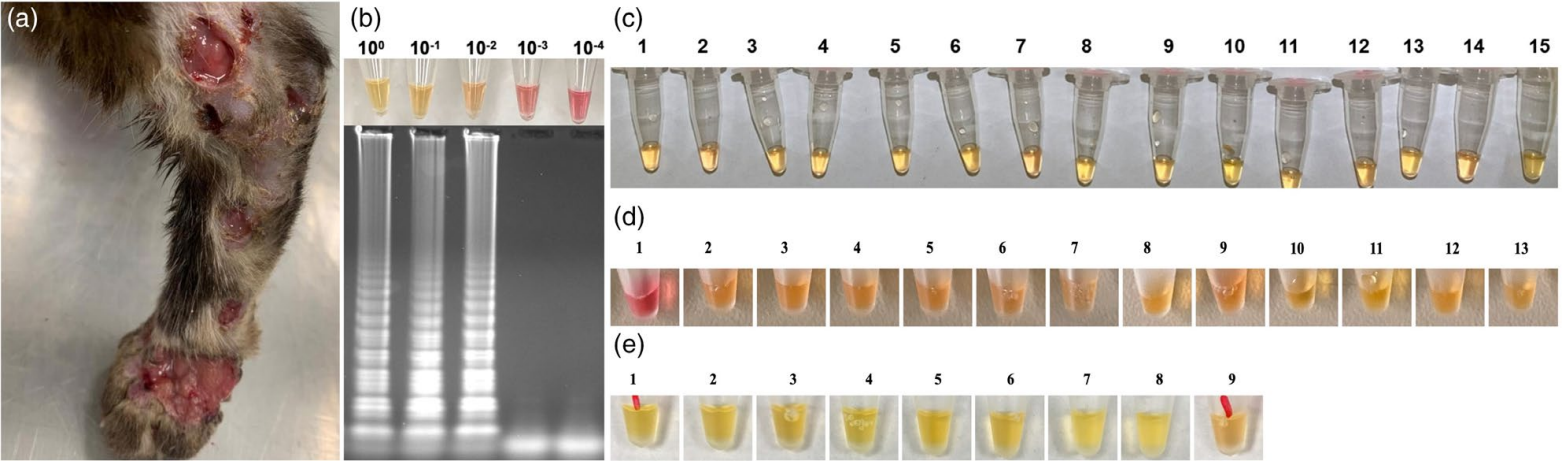
*Sporothrix* detection in blood and cutaneous lesions from feline clinical samples. (a) A cat with sporotrichosis presenting characteristic cutaneous lesions. (b) Limit of the colorimetric loop‐mediated isothermal amplification (LAMP) assay detection using *Sporothrix* DNA templates (ranging from 1 to 10^−4^ ng) in a blood matrix, along with gel electrophoresis analysis of the LAMP assay products. (c) Colorimetric LAMP assay results for blood samples collected from cats positive for sporotrichosis, targeting the 28S ribosomal RNA region of *S. brasiliensis* and *S. schenckii*. (d) Colorimetric LAMP assay results for swab samples collected from cutaneous lesions of cats positive for sporotrichosis. (e) Colorimetric LAMP assay results for adhesive tape samples collected from cutaneous lesions of cats positive for sporotrichosis. Colour interpretation: pink represents negative reactions, while yellow or orange indicates positive LAMP amplification.

**FIGURE 3:**
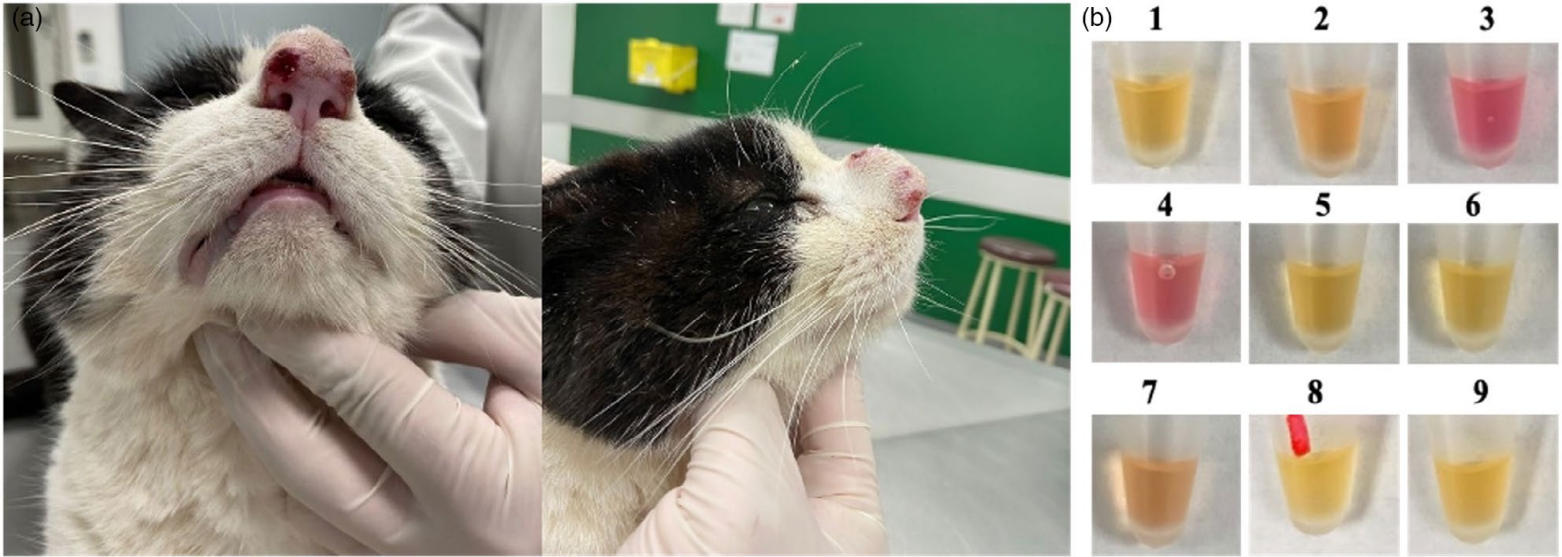
*Sporothrix* detection in nasal discharge samples from feline clinical cases. (a) A cat diagnosed with sporotrichosis exhibiting characteristic nasal lesions associated with the disease. (b) Colorimetric loop‐mediated isothermal amplification (LAMP) assay results for intranasal discharge samples collected from cats positive for sporotrichosis. Colour interpretation: pink represents negative reactions, while yellow or orange indicates positive LAMP amplification.

The LOD for the spiked LAMP assay was 10 pg of *Sporothrix* DNA in the blood matrix (Figure 2b). Then, 46 samples collected directly from the patients' fluids positive for sporotrichosis were submitted to amplification using the LAMP technique. Regarding blood samples, all 15 were positive for *Sporothrix* using the LAMP technique (Figure 2c), resulting in a sensitivity of 100%. Minimally invasive sampling of cutaneous lesions demonstrated high sensitivity rates: 92.31% (12 of 13) for swabs (Figures 2a,d) and 100% (9 of 9) for adhesive tape samples (Figure 2e). Nasal mucosa infection (Table 2; Figure 3a) demonstrated 77.78% (7 of 9) sensitivity using intranasal swabs (Figure 3b). False‐negative results were observed in two intranasal swab samples and one cutaneous lesion swab. However, all of these false‐negative samples successfully amplified the feline internal control. Considering the results, the LAMP assay successfully detected *Sporothrix* DNA targeting the 28S sequence from mycological isolates, blood, intranasal and cutaneous lesion samples.

**TABLE 2:** Comparison of detection methods for *Sporothrix* detection in feline clinical samples.

ID	Antifungal treatment	Infection site	Lesions	Fungal culture	Cytological results	LAMP result per sample		
						Intranasal swab	Cutaneous swab	Cutaneous tape
6301	POS	Nasal mucosa	Multiple	POS	POS	POS	–	–
6302	POS	Limbs	Multiple	POS	POS	–	POS	–
6303	POS	Nasal mucosa	Multiple	POS	POS	–	POS	–
6304	POS	Ocular adnexal	Multiple	POS	POS	–	POS	–
6305	POS	Nasal mucosa	Multiple	POS	POS	POS	–	–
6306	POS	Nasal mucosa	Multiple	POS	POS	POS	–	–
6307	POS	Ear pinna	Multiple	POS	POS	–	POS	–
6308	POS	Face, limbs	Multiple	POS	POS	–	POS	POS
6336	POS	Limbs	Multiple	POS	POS	–	POS	POS
6311	NEG	Limbs	Multiple	POS	POS	–	POS	POS
6312	NEG	Face	Multiple	POS	POS	POS	POS	POS
6313	NEG	Nasal mucosa	Multiple	POS	NEG	POS	POS	POS
6467	NEG	Limbs	Multiple	POS	NA	POS	POS	POS
6465	NEG	Nasal mucosa	Multiple	POS	NA	–	POS	POS
6300	POS	Nasal mucosa	Single	POS	POS	POS	–	–
6309	POS	Nasal mucosa	Single	POS	NEG	NEG	NEG	POS
6494	NEG	Nasal mucosa	Single	POS	NEG	NEG	POS	POS
Sensitivity				100%	80%	77.78%	92.31%	100%

Abbreviations: ID, identification; POS, positive; NEG, negative; NA, nonavailable.

## DISCUSSION

The present study introduced a 30‐min alternative for detecting *Sporothrix* from clinical samples using the LAMP assay. The assay demonstrated feasibility in identifying South America's primary *Sporothrix* species responsible for feline infections. Notably, the findings emphasise the usefulness of intranasal swabbing for detecting *Sporothrix* infections in cases of exclusive nasal manifestations when the standard fungal culture is unsuitable. As a consequence of its simplicity and practicality, the LAMP assay shows promise as a tool for routine use in veterinary clinics, providing a convenient and effective screening method.

Other studies detecting sporotrichosis using LAMP corroborated the findings of the present study, such as the study by Chupia et al., which identified *S. schenckii* ^34^ and that of Fan et al., which identified *S. globosa*.^35^ The analysis of isolated specimens indicates that targeting the 28S region could effectively detect clinically relevant *Sporothrix* species, ensuring broad inclusivity in the screening test. This study also found that the assay's detection limit ranged from 1 to 10 pg of *Sporothrix* DNA. The LAMP assay spiked with *Sporothrix* in the blood matrix showed a LOD of 10 pg of DNA (per 25 μL of reaction), a result similar to that of Chupia et al.,^34^ who investigated the use of LAMP for identifying *S. schenckii* and reported a LOD of 10 pg targeting the 18S rRNA gene. This LAMP technique showed less sensitivity in spiked samples than in water, which is likely to be a result of inhibitors present in blood and the EDTA anticoagulant used in the blood collection tube.^36^ Notwithstanding this, LAMP achieved LODs comparable to other molecular techniques for detecting *Sporothrix*. It demonstrated similar sensitivity to PCR and quantitative (q)PCR yet was less sensitive than a quantitative reverse transcription (qRT‐)PCR. For conventional PCR, Rodrigues et al.^16^ found a limit of 10–100 fg of DNA for identifying *S. brasiliensis*, *S. schenckii*, *S. globosa, S. mexicana* and *S. pallida* targeting calmodulin gene sequences; Liu et al.^12^ found 5 pg for *S. schenckii*; and Rodríguez‐Brito et al.^13^ detected 200 fg of *Sporothrix* DNA using a qPCR and 20 pg with a PCR targeting the 18S rRNA gene. In other qPCR studies, Zhang et al.^37^ reported a LOD of 10 fg of DNA for *S. globosa* by targeting the *Sporothrix* internal transcribed spacer; and Almeida‐Silva et al.^38^ found a LOD of 10^−2^ pg of *S. brasiliensis* DNA targeting calmodulin gene sequences. A qRT‐PCR study identifying the *Sporothrix* pathogen clade found a LOD of 244 fg.^39^ The LOD can vary depending on the protocol, target regions and probes. Notably, LAMP may outperform conventional PCR in sensitivity because DNA amplification inhibitors have a greater impact on PCR at lower concentrations than LAMP assays.^40^

Molecular techniques are powerful tools for detecting *Sporothrix* DNA, yet they are underutilised in direct clinical diagnostics. Most current studies focus on detecting *Sporothrix* DNA from pure fungal cultures rather than directly from clinical samples,^39^, ^41^, ^42^ limiting their practical application in real‐world settings. Therefore, standardised protocols are needed to detect *Sporothrix* DNA from clinical samples. Cutaneous lesions showed sufficient DNA, reflecting a high sensitivity when using swabs and adhesive tape for LAMP assay. This result may be the consequence of a high fungal load in nodules or ulcers in feline skin, which could be higher than in the nose — corroborating Schubach et al.'s study that isolated *S. schenckii* in 100% of cutaneous lesions, in 66.2% of nasal cavities and in 41.8% of oral cavities of cats with sporotrichosis.^43^ In feline sporotrichosis, extracutaneous signs, particularly respiratory ones, are frequent. This occurrence may precede the appearance of cutaneous lesions,^44^ being hypothesised that the cats' infection also may occur through inhalation.^43^, ^45^ One of the treatment challenges in cats with sporotrichosis is nasal infection.^46^ These cases have been associated with poor responses to antifungal treatment, resulting in reoccurrence even after significant improvement in clinical signs^47^, ^48^, ^49^ and associated with treatment failure and death.^49^, ^50^ Despite the importance of detecting and monitoring sporotrichosis in extracutaneous forms, the fungal culture is unsuitable for nasal cases. Therefore, this study detected *Sporothrix* DNA from cases with nasal signs exclusively and supports a new alternative to clarify the prevalence of extracutaneous manifestations, particularly respiratory signs, in feline sporotrichosis.

Antifungal treatment of patients did not influence the LAMP results. Interestingly, animals with single lesions in the nasal mucosa were more likely to yield negative results for both LAMP and cytological testing, which may indicate a lower fungal load in these localised infections. The false‐negative patient samples for 28S LAMP still presented the feline internal control, suggesting that the absence of *Sporothrix* DNA is unlikely to be an artefact of sample collection or DNA extraction issues. Instead, these results are likely to reflect that the fungal load in these lesions was below the LOD of the LAMP assay; this may indicate an early stage of infection or a less aggressive fungal growth, which could be more challenging to detect in single, localised lesions compared to cases with multiple lesions or more advanced infections, where higher fungal loads are more likely to be present in various clinical samples.

The 28S target LAMP assay presents a promising approach for screening sporotrichosis, yet the study did not evaluate its performance in different clinical samples of cats negative for sporotrichosis. This limitation may affect the specificity of the LAMP assay, highlighting the need for future studies incorporating characterised negative specimens to provide a more comprehensive evaluation. True‐negative specimens should be characterised using a standardised molecular method, such as PCR, for comparative analysis. Notably, the absence of fungal growth does not confirm a true‐negative patient, leaving the possibility of a false‐negative sample. Also, primer selectivity was assessed only through Blast in silico analyses, which may not represent a LAMP amplification. These algorithms check the cross‐homology between primer pairs and the database,^27^ yet do not account for secondary structures formed by Loop‐F/Loop‐B and FIP/BIP during LAMP amplification. Additionally, the study is limited to *L. infantum* and *M. leprae* for nontarget DNA analysed in vitro. Thus, other cat skin‐related diseases which may present with clinical signs similar to sporotrichosis, such as cutaneous leishmaniosis and mycobacterial infection, should be tested to explore further the selectivity of LAMP primer.

Hence, this study suggests that the LAMP assay has potential as a quick, routine screening tool in veterinary clinics, offering an alternative approach to the early detection of *Sporothrix* in challenging clinical settings. The ability to detect *Sporothrix* directly from clinical samples, including those with respiratory involvement, could support the disease investigation when fungal culture analysis might be unsuitable. Overall, the LAMP assay is a reliable, sensitive and noninvasive diagnostic tool for detecting *Sporothrix* DNA from various clinical samples, with strong potential for use in veterinary diagnostics.

## AUTHOR CONTRIBUTIONS

**Steffanie Amadei:** Conceptualization; investigation; funding acquisition; writing – original draft; writing – review and editing; methodology; visualization; project administration. **Júlia Campos:** Investigation; validation; visualization. **Amanda BERTÃO‐SANTOS:** Investigation; methodology; validation. **Alis Frentzel:** Conceptualization; funding acquisition; investigation; methodology. **Hugo Ávila:** Funding acquisition; data curation; software; formal analysis. **Fabiana S. Monti:** Supervision; resources; project administration. **Marconi R. Farias:** Supervision; resources; funding acquisition; conceptualization; methodology; writing – review and editing; project administration.

## FUNDING INFORMATION

Conselho Nacional de Desenvolvimento Científico e Tecnológico. Fundação Araucária. Pontificia Universidade Católica do Paraná.

## CONFLICT OF INTEREST STATEMENT

The authors declare no conflicts of interest.

## Data Availability

The data that support the findings of this study are available from the corresponding author upon reasonable request.
